# Self-Powered Speed Sensor for Turbodrills Based on Triboelectric Nanogenerator

**DOI:** 10.3390/s19224889

**Published:** 2019-11-09

**Authors:** Chuan Wu, Chenxing Fan, Guojun Wen

**Affiliations:** 1 Faculty of Mechanical and Electronic Information, China University of Geosciences (Wuhan), Wuhan 430074, China; fancx@cug.edu.cn; 2 Key Laboratory of Metallogenic Prediction of Nonferrous Metals and Geological Environment Monitoring, Ministry of Education, Central South University, Changsha 410012, China; 3 Chair for Mechanics and Robotics, University of Duisburg-Essen, Duisburg 47057, Germany

**Keywords:** speed sensor, triboelectric nanogenerator (TENG), turbodrills, self-powered

## Abstract

Turbodrills play an important role in underground energy mining. The downhole rotational speed of turbodrills is one of the key parameters for controlling the drilling technology. Therefore, it is necessary to measure the rotational speed of the turbodrills in real time. However, there is no dedicated speed sensor for the working environment of turbodrills at present. Therefore, based on the working principle of triboelectric nanogenerator (TENG), a self-powered speed sensor which can measure the speed of the turbodrills is proposed in this study. Besides, since the sensor is self-powered, it can operate without power supply. According to the laboratory test results, the measurement error of the sensor is less than 5%. In addition, the self-powered performance of the sensor was also explored in this study. The test shows that the maximum generating voltage of the sensor is about 27 V, the maximum current is about 7 μA, the maximum power is about 2 × 10^−4^ W, and the generated electricity can supply power for ten LED (light-emitting diode), which not only meets the power supply requirements of the sensor itself, but also makes it possible to further power other underground instruments.

## 1. Introduction

As a kind of fluid machine used in drilling, the turbodrills relies on the impact of fluids to generate a rotary motion for rock fragmentation to achieve well drilling. The rotational speed of a turbodrills when working underground is not only an important parameter for calculating its output power and efficiency, but also a significant basis for controlling the on-site drilling process. Hence, it is necessary to measure the downhole rotational speed in real time. The measurement of the rotational speed is widely applied in the detection of industrial systems at present. Li et al. [1] proposed a digital electrostatic sensor, which measures the period and frequency of the signals to achieve the purpose of rotational speed measurement, with the measurement error being less than 4%. Lin et al. [2] proposed a codeless speed sensor based on the vibration signal and improved the measurement accuracy with the discrete spectrum correction technology. Based on the principle of rotating magnetic field, Arif et al. [3] detected the change of the rotational speed within 2.5 ms. Mirzaei et al. [4] designed a new type of non-contact eddy current speed sensor when the speed is lower than 1200 r/min. As for the problem that conventional sensors cannot be mounted on a rotating shaft, Zhong et al. [5] measured the rotational speed based on the stripe vision system. Pawlenka et al. [6] measured the rotational speed of high-speed objects using Kalman filtering, achieving certain results. However, restricted by the actual working conditions (e.g., high temperature, high pressure, and strong vibration underground), the current measurement technology is unable to meet the needs of downhole rotational speed measurement. The triboelectric nanogenerator, first proposed in 2012 [7], had achieved wide application in the field of sensors [8,9,10], and is expected to solve the problem of downhole vibration measurement. In addition, since the output voltage value of the triboelectric nanogenerator changes with high temperature is not obvious [11], the sensor can theoretically adapt to the high temperature environment of the deep well, and it is expected to be further developed as a high-temperature downhole sensor. Therefore, a speed sensor for the turbodrills based on triboelectric nanogenerator was proposed in this study and the sensor is self-powered, which means it can work without external power supply.

## 2. Design of the Sensor

### 2.1. Theoretical Basis of the Sensor

The sensor is designed based on the TENG. The working principle of TENG is triboelectrification [12,13], that is, when the two materials rub against each other, electric charge will be generated on the surface of the material because of triboelectrification, and when the two materials separate from each other, current transfer will occur between the two materials. So, when the friction process occurs in circles, that is, when the two materials keep contacting and separating from each other, the charge on the surface of the materials will be transferred regularly, so the downhole speed sensor for the turbodrills can be made according to this principle. Based on this, the basic principle of the designed sensor is as follows: When the turbodrills rotates, the turbodrills drives the two materials inside the sensor to achieve periodic “contact-separation.” At this time, because of triboelectrification, the periodic “contact-separation” will lead to regular charge transfer, that is to say, the charge transfer law is proportional to the turbodrills speed. So, the rotational speed can be obtained by using hardware circuit to further measure the charge transfer law. In addition, since the electric energy is generated during the triboelectrification process, the self-powered sensor can work without power supply.

### 2.2. Structure of the Sensor

**Figure 1:**
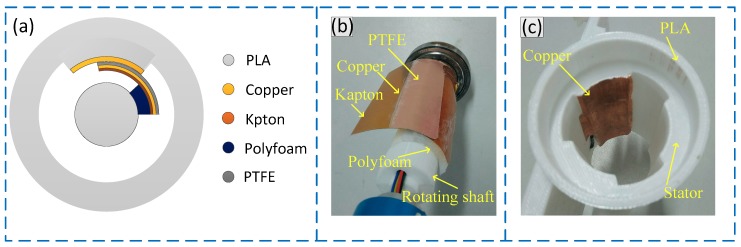
Schematic diagram of the sensor structure. (**a**) Schematic diagram of the overall structure; (**b**) picture of rotating shaft of the sensor; (**c**) picture of the sensing stator.

Figure 1 shows the schematic diagram of the structure of the sensor. Figure 1a shows the overall structure and composition of the sensor. The sensor consists of the rotating shaft and the stator. The rotating shaft is connected to the support section of the turbodrills and keeps rotating while the stator is connected to the turbine section of the turbodrills and keeps stationary. A layer of friction material is applied to both the rotating shaft and the stator. When the turbodrills is working, the friction material on the rotating shaft and the stator features a periodic process of “contact-separation,” achieving the measurement of the rotational speed based on the charge transfer law during the “contact-separation” process. Figure 1b,c presents the rotating shaft and the stator of the sensor respectively. The main structure of the sensor was 3D printed with the PLA material. The PLA material need a printing temperature of 210 °C, a blank thickness of 0.2 mm, and a structural duty ratio of 60%. A layer of copper material with a thickness of 0.05 mm was applied as a friction material on the inner wall of the stator, which also functions as an electrode for deriving the generated electric charge. The PTFE material (CTF30, Bench Co., LTD, Suzhou, China.) with a thickness of 0.03 mm was applied to the outer wall of the rotating shaft, and a layer of copper material, as another electrode, was attached on the back surface of the PTFE material (near the direction of the rotating shaft) for deriving the generated electric charge. Since the PTFE material and the copper material are relatively soft, problems such as entanglement may occur at a high rotational speed, which will affect the frictional electrification process. Therefore, the 0.1-mm-thick Kapton material with relatively good elasticity was adhered to the back surface of the copper material to support the PTFE material and copper material, ensuring the contact area between the rotating shaft and the stator. Moreover, based on the shrinkage of the foam, the Kapton material served as a buffer layer to convert the hard friction into soft friction, reducing the wear of the friction surface and improving the service life.

### 2.3. Working Principle of the Sensor

**Figure 2:**
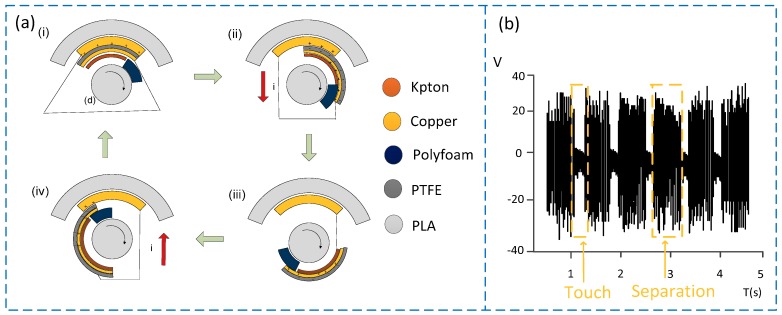
Working principle of the sensor. (**a**) Composition and working principle of the sensor; (**b**) signal waveform of the sensor.

According to the above description of the sensor structure, when the sensor is working, the copper material and the PTFE material are in contact with each other regularly. Because of the triboelectrification, charge transfer will occur in the part where the two materials contact. According to the materials’ abilities to obtain and lose electric charge, the copper material is more likely to lose electrons than the PTFE material [14], because of the highest percentage of fluorine in PTFE, which leads to strong electron attraction. In addition, since the nucleus of copper is less attractive to external electrons, it is easy to lose electrons. So the surface of the copper electrode is positively charged while that of the PTFE material is negatively charged. Since the polymer material has good insulating property, the charge can remain on the surface of the material for a long time without disappearing [15]. Figure 2 shows the working principle of the sensor. When the sensor is working, the contact state between the rotating shaft and the stator inside the sensor can be divided into four processes, i.e., coincidence, relative slip, complete separation, and re-contact (as shown in Figure 2a). Specifically, as shown in Figure 2a(i), the two materials are in contact with each other to generate electric charge and charges with opposite polarities are in a state of mutual balance. At this time, no electric charge flows in the external circuit and the voltage approaches zero. The process of relative slip is shown in Figure 2a(ii), where there is a relative slip between the two materials because of the rotation of the rotating shaft, and the electric charges in the staggered position cannot cancel each other, thereby destroying the balanced state reached before. According to the electrostatic induction effect, in order to balance the potential in this area, the charge between the two electrodes will be transferred until the potential in this area is balanced again. Thus, when there is constant relative slip between the two friction materials, charge transfer continues between the two electrodes, with the voltage value gradually increasing. Figure 2a(iii) shows the state of complete separation. At this time, since the two friction materials are not in contact, there is no charge transfer between the electrodes, but the voltage between the two electrodes reaches the maximum saturation value. As the rotating shaft continues to rotate, it is the re-contact state as shown in Figure 2a(iv). When the PTFE friction layer and the copper material contact again, the charge will be transferred in the opposite direction and the voltage will gradually decrease until the two layers return to the state of complete bonding. Figure 2b shows the signal waveform of the sensor when it is working. When the two materials are in contact, the voltage decreases and approaches zero. When the two materials are not in contact, the voltage reaches a saturation value. It can be seen from the above description that the direction of charge transfers changes once in a cycle, that is, the alternating current signal output is generated, and the signal frequency is proportional to the rotational speed, so the frequency of the periodic signal can be counted through the hardware circuit, thereby realizing the measurement of the rotational speed.

## 3. Experiment

### 3.1. Experimental Facilities

**Figure 3:**
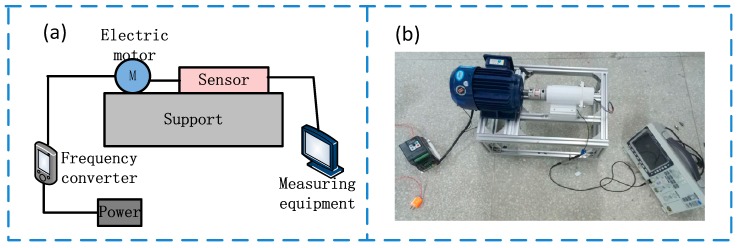
Schematic diagram of experimental facilities. (**a**) Composition of experimental facilities; (**b**) physical picture of experimental facilities.

Figure 3 shows the schematic diagram of the experimental facilities. The motor can output different rotational speeds under the regulation of the inverter to simulate the speed output of the real turbodrills. The sensor and the motor are on the same level. In order to simulate the sensor’s installation position in the real turbodrills, the output shaft of the motor drives the sensor’s rotating shaft to rotate, while the stator is fixed. The signals generated by the sensor are read by the GDS-3152 oscilloscope (Goodwill Co., LTD., New Taipei City, Taiwan), and the rotational speed is calculated according to the frequency of the output signal.

### 3.2. Rotational Speed Measurement

**Figure 4:**
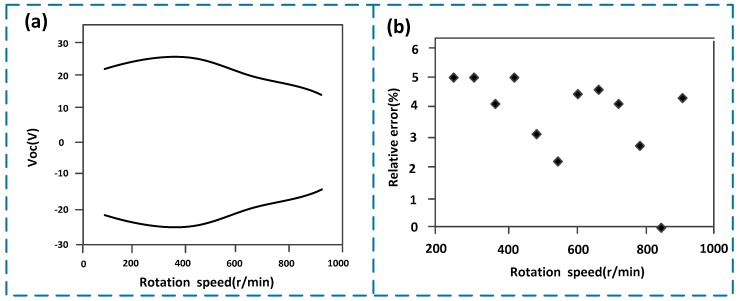
Statistical analysis results of the measured data under different rotational speeds. (**a**) Signal voltage amplitude of the sensor under different rotational speeds; (**b**) scatter diagram of relative error of the measured rotational speed.

Figure 4 shows the statistical analysis results of the measured data under different rotational speeds. The signal voltage amplitude of the sensor under different rotational speeds are shown in Figure 4a, where the abscissa represents the actual speed and the ordinate represents the envelope of the output signal voltage amplitude. As shown in Figure 4a, when the rotational speed is around 400 r/min, the average voltage amplitude generated by the sensor is about 27 V, and at the rotational speed of about 900 r/min, the average voltage amplitude generated by the sensor is about 17 V. In other words, there is an inverse proportional relationship between the rotational speed and the signal amplitude within a specific area. The reason is that at a low rotational speed (the speed is around 400 r/min), the time interval between the two internal frictions inside the sensor is relatively long, and the material have enough time to return to its initial state after friction deformation, which guarantees the friction area. Since a larger friction area means a larger amount of charge generated, the output voltage is comparatively large as well. On the contrary, when the speed is high (the speed is around 900 r/min), the time interval between the two frictions is relatively short, so there is no enough time for the deformed materials to return to its initial state. Consequently, the frictional contact area is reduced, because of which the amount of charge generated decreases, so that the output voltage is comparatively small. Figure 4b shows the scatter diagram of relative error of the measured rotational speed according to the statistical analysis to 100 times of test data. The abscissa represents the actual speed, and the ordinate stands for the relative error of the measured speed. According to Figure 4b, the measurement error is between 2% and 5% (i.e., the maximum measurement error of the sensor is less than 5%), and there is no significant linear correlation between the error distribution and the rotational speed. This error is acceptable in the actual drilling process.

### 3.3. Self-Powered Experiment

The working principle of the sensor is triboelectrification, so the sensor can work normally without power supply. In addition, if the electric energy generated by the sensor is collected and stored, can the electric energy power other underground instruments when the power reaches a certain value? Hence, the self-powered performance of the sensor was explored in this study.

**Figure 5:**
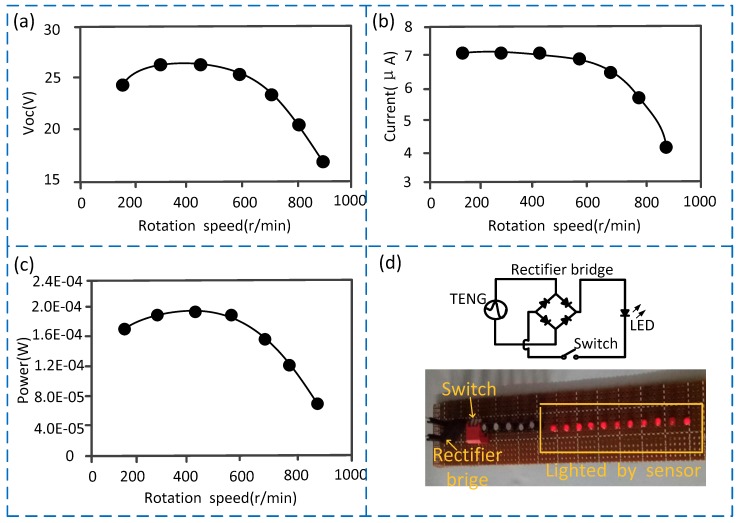
Experimental results of the self-powered sensor. (**a**) Curve showing the relationship between the output voltage and the rotational speed; (**b**) curve showing the relationship between the output current and the rotational speed; (**c**) curve showing the relationship between the output power and the rotational speed; (**d**) a picture shown the LED (light emitting diode) lighted by the sensor’s output power.

The frictional electrification parameters of the sensor include voltage, current, and power. Therefore, based on the statistical analysis of the 100 times test data, we separately obtained the relationship between the frictional electrification parameters and the rotational speed (as shown in Figure 5). Figure 5a shows the relationship between the output voltage and the rotational speed. The abscissa represents the actual speed, with the unit being RPM (r/min), and the ordinate represents the voltage amplitude generated by the sensor, with the unit being volt (V). Figure 5b shows the relationship between the output current and the rotational speed. The abscissa represents the actual speed, with the unit being RPM (r/min), and the ordinate represents the current amplitude generated by the sensor, with the unit being microampere (μA). Figure 5c shows the relationship between the output power and the rotational speed. The abscissa represents the actual speed, with the unit being RPM (r/min), and the ordinate represents the power generated by the sensor, with the unit being watt (W). The test facilities used in Figure 5 are also as shown in Figure 3, and the output voltage, current, and power are measured by electrometer (6514, Keithley Co., LTD, Solon, OH, USA). 

As shown in Figure 5, the output voltage, the current, and the power all increase first and then decreases slowly with the increase of the rotational speed. To the output voltage, the maximum value is about 27 V when the rotational speed is about 400 r/min, and the minimum value is about 17 V when rotational speed is about 900 r/min. To the output current, the maximum value is about 7 μA when the rotational speed is below about 600 r/min, and the minimum value is about 4 μA when the rotational speed is around 900 r/min. To the power, the maximum value is about $2\times 10^{-4}\;W$ when the rotational speed ranges from 200 r/min to 600 r/min, and the minimum value is about $6\times 10^{-5}\;W$ when the rotational speed is about 900 r/min. 

Furthermore, to further visually display the self-powered performance of the sensor, the alternating current is converted to direct current through the rectifier bridge, and we directly supplied multiple LED lights with the power output by the sensor. The number of lighted LED lights implies the real-time power supply effect. According to the test, directly driven by the sensor, the maximum power generated can continuously light 10 LED lights (Figure 5d). If multiple sensors are connected in series and the generated power is stored in a battery for a certain period of time, it is possible to supply power in real time for the downhole low-power instruments, such as the measuring while drilling (MWD) instruments.

## 4. Conclusions

1. A self-powered speed sensor for the turbodrills based on triboelectric nanogenerator was proposed in this study. According to a statistical analysis of the test data, the measurement range is 0 to 900 r/min and the measurement error is less than 5%, which can meet the actual drilling demand, but the measurement error is slightly larger. So how to improve the sensor structure to further improve the measurement accuracy is a key point that needs to be further studied.
2. The output signal voltage value reaches a peak value of about 27 V at lower speeds. The larger of the output signal voltage value, the better to the detection of the signal. Therefore, the sensor has better signal characteristics at low speeds.
3. The sensor is self-powered, which means that it can work without power supply. The self-powered performance of the sensor was studied in this study and the test results shown that the maximum voltage is about 27 V, the current is about 7 μA, and the power is about $2\times 10^{-4}\;W$.
4. In addition to meeting its own power supply requirements, the sensor’s output power can also be stored to supply other downhole instruments. Nonetheless, the power is currently small. So, how to increase the power generation by comprehensively considering the selection, the processing, and the surface characteristics of the nanomaterials is another key point that needs to be further studied.
